# Acute Neurological Deficit With New-Onset Atrial Fibrillation in a Patient With Carbon Monoxide Poisoning: A Stroke Mimic

**DOI:** 10.7759/cureus.93283

**Published:** 2025-09-26

**Authors:** Falaah Abdul Hameed, Mohammed Abdulwahab Ali Farid Al Ayafei, Phalguni Sai Preethi Asapu, Muneer Abdullah Almarzooqi

**Affiliations:** 1 Emergency Medicine, Sheikh Tahnoon Bin Mohammed Medical City, Al Ain, ARE; 2 Emergency Medicine, Tawam Hospital, Al Ain, ARE

**Keywords:** acute neurological deficit, carbon monoxide intoxication, clinical features of myocardial ischemia, electrocardiogram (ekg) atrial fibrillation (a-fib) with rapid ventricular response (rvr), hyperbaric oxygen therapy (hbot), nausea and vomiting, near syncope, oxygen therapy, smoking, stroke mimic

## Abstract

We present the case of a middle-aged male from the Middle East with no prior history of cardiac disease who presented with syncope, nausea, and dizziness following smoking and barbeque in the desert outdoors. On arrival, he was found to have atrial fibrillation (AF), with a heart rate of 150-170 bpm. A diagnosis of carbon monoxide (CO) poisoning was made based on venous blood gas (VBG) analysis, showing a carboxyhemoglobin level of 33.9%. Treatment was initiated with a non-rebreather mask, followed by high-flow nasal cannula therapy, which resolved the symptoms and arrhythmia within a day. Cardiology follow-up confirmed the complete regression of AF.

This case underscores uncommon but serious cardiac complications associated with CO poisoning. AF, although rare, may occur secondary to hypoxia-induced myocardial ischemia. Early recognition and prompt oxygen therapy are essential for optimal recovery, highlighting the importance of awareness when managing patients with CO poisoning with potential cardiac involvement.

## Introduction

Carbon monoxide (CO) poisoning is a significant global health concern, accounting for over half of all fatal poisoning cases worldwide. Inhalational injuries are implicated in more than two-thirds of fire-related deaths. Despite its profound impact, research on CO poisoning in the Middle East remains limited. CO exposure impairs oxygen delivery, predominantly affecting the brain and the heart. CO-induced hypoxia and oxidative stress may precipitate myocardial ischemia, arrhythmias, left ventricular dysfunction, and even sudden cardiac death in severe cases [1].

CO poisoning is a life-threatening condition with severe cardiac and neurological consequences. As a colorless, odorless gas, CO binds to hemoglobin with an affinity over 200 times that of oxygen, leading to tissue hypoxia and oxidative stress. Burning fuels, such as gas, wood, propane, and charcoal, produce carbon monoxide, which can accumulate at dangerous levels in poorly ventilated or enclosed spaces [2].

Stroke mimics represent a significant diagnostic challenge, accounting for approximately 20-30% of all suspected stroke presentations. Although the immediate symptoms of CO poisoning, such as headaches, dizziness, and confusion, are well documented, their long-term effects on the heart and brain remain a critical concern. CO-induced hypoxia and oxidative stress may precipitate myocardial ischemia, arrhythmias, left ventricular dysfunction, and even sudden cardiac death in severe cases. Neurological damage can manifest as cognitive impairment, memory deficits, and delayed neuropsychiatric syndrome. This case report highlights the dual impact of CO poisoning on both the cardiac and neurological systems, emphasizing the need for early recognition, rapid intervention, and long-term follow-up to mitigate lasting morbidity [3].

## Case presentation

A 36-year-old male with no known comorbidities presented to the emergency department with nausea, vomiting, dizziness, and a syncopal episode. History obtained from the patient and his friends revealed that he had been smoking shisha for nearly two hours while grilling a barbecue in an open desert setting, sitting close to the fire. He denied drinking beverages, including alcohol and caffeinated beverages, or ingesting any other substances. 

**Figure 1:**
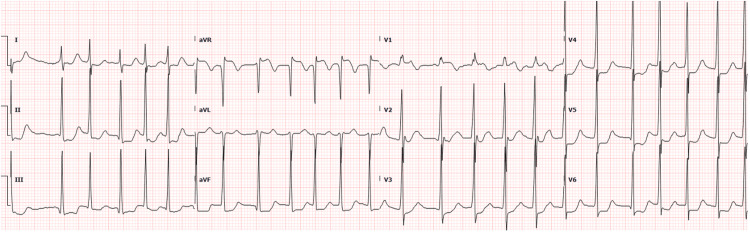
Initial electrocardiogram showed atrial fibrillation with widespread ST segment depression and ST segment elevation in avR.

**Figure 2:**
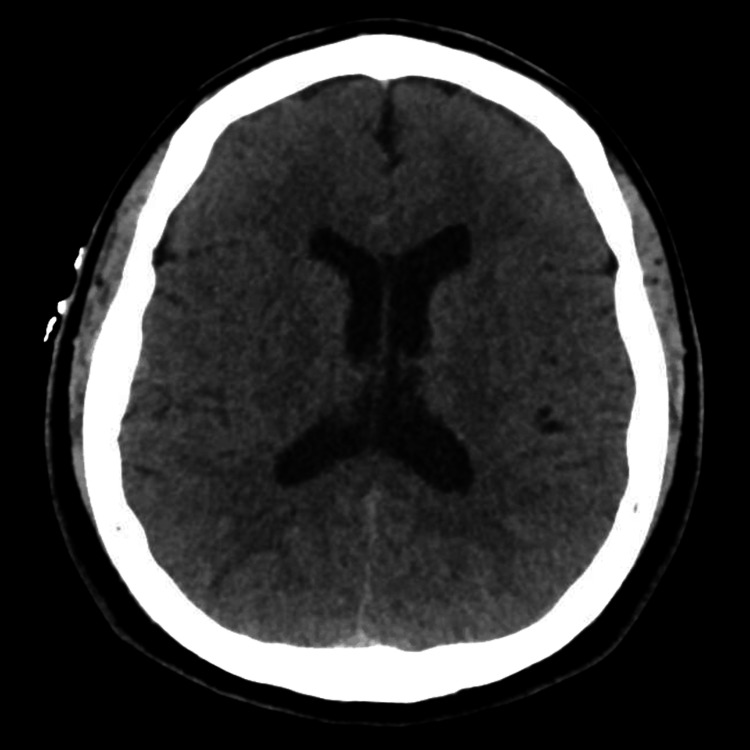
Computed tomography scan of the head showed no abnormalities.

Upon arrival via ambulance, the patient was alert but continued to experience dizziness and nausea. His vital signs included a heart rate of 150-170 bpm with an irregular rhythm, respiratory rate of 20 bpm, blood pressure of 126/104 mmHg, and temperature of 36.6°C. Physical examination was unremarkable, and neurological assessment revealed no sensory or motor deficits, speech difficulties, nystagmus, or ataxia, aside from an initially unsteady gait. Electrocardiography (ECG) confirmed atrial fibrillation (AF) with widespread ST depressions and ST segment elevation in avR (Figure 1), and venous blood gas (VBG) analysis identified a CO level of 33.9% and a low potassium level of 2.2 (Table 1). A stroke code was activated from triage, but the subsequent CT scan of Head and Neck was unremarkable (Figure 2).

**Table 1:** Laboratory Investigations: Blood Chemistry, CBC and Coagulation eGFR: estimated glomerular filtration rate, MCV: mean corpuscular volume, MCH: mean corpuscular hemoglobin, MCHC: mean corpuscular hemoglobin concentration, RDW-CV: red cell distribution width coefficient of variation, MPV: mean platelet volume, PT: prothrombin time, INR: international normalised ratio, aPPT: activated partial thromboplastin time

General Chemistry		
Parameter	Value	Reference Range
Sodium	142	136–145 (mmol/L)
Potassium	2.2	3.2–5.5 (mmol/L)
Chloride	114	98–107 (mmol/L)
CO2	17	22–29 (mmol/L)
Creatinine	56	62–106 (µmol/L)
Urea	3.53	2.8–8.1 (mmol/L)
Glucose (Random)	6.6	3.9–7.8 (mmol/L)
Troponin-T	7.0	≤14.0 (ng/L)
eGFR (CKD-EPI)	126	≥60 (mL/min)
Complete Blood Count		
WBC	12.2	5.5–11 (×10⁹/L)
RBC	4.99	4.30–5.70 (×10¹²/L)
Hemoglobin (Hgb)	133	132–173 (g/L)
Hematocrit (Hct)	0.38	0.39–0.49 (L/L)
MCV	75.6	80.0–99.0 (fL)
MCH	26.7	27.0–34.0 (pg)
MCHC	353	320–370 (g/L)
Platelet Count	340	140–400 (×10⁹/L)
RDW-CV	14.30	11.6–14.8 (%)
MPV	9.1	9.6–12 (fL)
Coagulation Profile		
PT	12.1	9.5–12.5 (sec)
INR	1.13	0.87–1.15
APTT	22.8	22.2–34.2 (sec)

Additionally, laboratory test results, including random blood sugar, hemoglobin, and troponin T levels, were all within normal limits (Table 1).

The patient was initially managed with a non-rebreather mask and later transitioned to a high-flow nasal cannula (HFNC). He received potassium chloride infusion, 5 mg IV metoprolol and 2 liters of intravenous fluids. The poison control center was consulted, and it was advised to administer 100% oxygen therapy for five to six hours while continuing observation.

**Figure 3:**
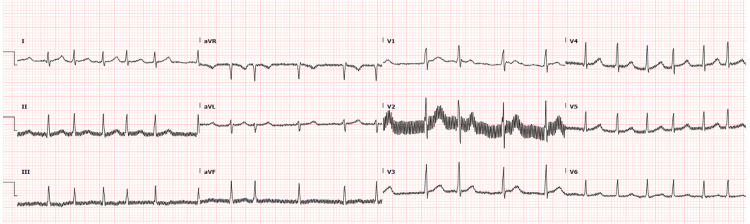
Repeated electrocardiogram after four hours still showed atrial fibrillation.

After four hours of treatment, the patient’s symptoms resolved. However, repeat ECG still showed atrial fibrillation, with a heart rate of 121 bpm (Figure 3). Repeat VBG showed a CO level reduction to 4% with a potassium level of 4.3mmol/L (Table 2).

**Table 2:** Venous Blood Gas Analysis: Initial and Four-Hour Comparison BE: base excess, Hgb: hemoglobin, O_2_HB: oxyhemoglobin, COHB: carboxyhemoglobin, MetHb: methemoglobin

Parameter	Initial Value	Value at 4 Hours	Reference Range
pH	7.36	7.34	7.35–7.45
pCO₂	48.2	52.2	35.0–45.0 (mmHg)
pO₂	11.4	32.8	25.0–40.0 (mmHg)
HCO₃ (Act)	27	28	22–26 (mmol/L)
BE	0.7	1.3	(−2.0)–2.0 (mmol/L)
Total Hgb	143	143	120–170 (g/L)
O₂ Saturation	23.60	60.60	95.0–99.0 (%)
O₂HB	15.40	57.60	≥60 (%)
COHB	33.90	4.00	0.0–2.0 (%)
MetHb	1.00	0.90	0.0–1.5 (%)
Potassium	3.2	4.3	3.4–5.1 (mmol/L)
Chloride	103	103	98–107 (mmol/L)
Sodium	143	142	136–145 (mmol/L)
Glucose	6.6	5.1	3.9–6.0 (mmol/L)
Ionized Calcium (iCa)	1.15	1.20	1.12–1.32 (mmol/L)
Lactate	3.8	3.7	0.5–2.2 (mmol/L)

The patient was also noted to have significant hypokalemia (serum potassium 2.2 mmol/L), which could have contributed to the development and persistence of atrial fibrillation. Hypokalemia is a well-recognized risk factor for cardiac arrhythmias due to its effects on myocardial excitability and conduction. While CO-induced hypoxia and myocardial stress likely played the primary role, the concurrent electrolyte disturbance may have acted as an additional trigger. This highlights the importance of evaluating and correcting metabolic abnormalities in patients presenting with arrhythmias, especially in the context of toxic exposures where multiple factors play a role.

**Figure 4:**
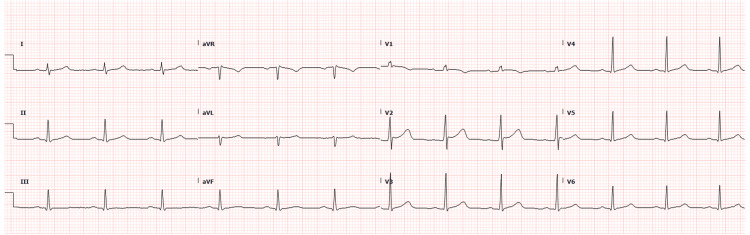
Electrocardiogram showed Sinus rhythm on admission to Internal Medicine.

The patient was admitted to an internal medicine telemetry unit for continued monitoring. As his heart rate remained uncontrolled, bisoprolol 5 mg every 12 hours was initiated per the cardiology recommendation. The sinus rhythm was restored on the day of admission (Figure 4). Echocardiography (ECHO) and thyroid function test results were normal. The patient was discharged after three days with instructions to follow up in an outpatient cardiology clinic and continued bisoprolol for one month.

At the two-week cardiology and internal medicine clinic follow-up, the patient reported feeling well and was asymptomatic. A repeat ECG confirmed a normal sinus rhythm. A final follow-up at 10 months showed that the patient remained symptom-free, with no recurrence of syncope, dizziness, palpitations, or other complaints. 

## Discussion

This case highlights the dual impact of CO poisoning on both the neurological and cardiovascular systems. The patient presented with an acute neurological deficit that initially raised concern for stroke but was ultimately found to have new-onset AF secondary to CO poisoning. This underscores the importance of considering CO poisoning in patients with acute neurological and cardiac symptoms, particularly those with a potential exposure history [4].

CO poisoning exerts toxic effects primarily through hypoxia, oxidative stress, and inflammation, leading to myocardial ischemia and neurological dysfunction [4]. While neurological manifestations, such as confusion and dizziness, are well documented, cardiac complications (especially arrhythmias, such as AF) are less commonly reported but can have significant clinical implications. In this case, hypoxia-induced myocardial stress likely triggered AF, which was resolved with oxygen therapy and rate control measures. Similar instances have been documented in which CO exposure leads to cardiac arrhythmias, including AF, due to myocardial hypoxia and stress [5-7].

Several additional factors may have contributed to the onset of AF in this patient. Most notably, the initial laboratory investigations revealed significant hypokalemia (2.2 mmol/L), which was corrected with intravenous potassium supplementation. Although hypokalemia alone rarely causes AF, it is well recognized to lower the arrhythmogenic threshold. In combination with CO-induced hypoxia, this electrolyte disturbance may have facilitated the onset of AF. Moreover, shisha smoke contains not only CO but also nicotine and other toxic compounds. Nicotine is a sympathomimetic agent that increases catecholamine release and can promote arrhythmogenesis. Thus, the combined effects of CO-induced hypoxia, electrolyte disturbance, and nicotine-related sympathetic activation may have created a multifactorial substrate for AF in this patient [8].

Environmental factors may also have played a role. Given the desert climate, dehydration is a plausible contributing factor that can exacerbate electrolyte imbalance, reduce plasma volume, and increase cardiovascular strain. Additionally, it is important to emphasize that substantial CO exposure can occur even during shisha smoking in outdoor or semi-ventilated areas, as prolonged inhalation of large smoke volumes overwhelms the body’s ability to clear CO efficiently. This highlights the underestimated risks associated with shisha smoking even in seemingly safer environments [9].

Early recognition and prompt management are crucial for preventing severe complications of CO poisoning. VBG analysis played a pivotal role in identifying CO toxicity in this patient despite an initially unclear exposure history. This emphasizes the utility of VBG in acute settings, especially when clinical symptoms are nonspecific. Previous reports have highlighted VBG analysis as an essential tool for rapid diagnosis of CO poisoning, enabling timely intervention [7].

Hyperbaric oxygen (HBO) therapy is often considered in cases of severe CO poisoning to reduce the risk of long-term neurological sequelae [8]. However, its role in the prevention of cardiovascular complications remains unclear. In this case, the patient received standard oxygen therapy with complete resolution of symptoms and arrhythmia, raising the question of whether HBO would have provided additional benefits. Further research is needed to clarify the impact of HBO in preventing delayed cardiac or neurological complications associated with CO poisoning [10-14].

Long-term follow-up is essential because CO poisoning can have delayed sequelae affecting both the heart and the brain [14]. The patient remained asymptomatic, with no recurrence of AF or neurological deficits at one-year follow-up, suggesting a favorable outcome. Ongoing monitoring is recommended to detect late-onset complications. Cases have been reported in which patients developed delayed neuropsychiatric sequelae weeks after initial CO exposure, underscoring the need for prolonged observation [13,14].

This case highlights the critical need for increased awareness of the cardiac and neurological effects of carbon monoxide poisoning, the value of VBG analysis for early diagnosis, and the potential benefits of hyperbaric oxygen therapy in reducing long-term risks. Further research is essential to establish optimal management strategies for CO-induced cardiac arrhythmias and their long-term prognosis. 

## Conclusions

In conclusion, this case underscores that carbon monoxide poisoning can mimic acute stroke presentations and may be complicated by new-onset atrial fibrillation. The pathogenesis in this patient was likely multifactorial, with contributions from CO-induced hypoxia, significant hypokalemia, nicotine-related sympathetic stimulation, and possible dehydration, all of which may have lowered the arrhythmogenic threshold. While the patient recovered with supportive management, it is important to note that hyperbaric oxygen therapy, although not used in this case, is considered in many centers as a strategy to reduce delayed neurological and cardiovascular sequelae. This case emphasizes the importance of comprehensive evaluation including early venous blood gas analysis, correction of electrolyte abnormalities, and awareness of advanced therapies where available to optimize outcomes in CO poisoning. Further research is warranted to clarify the mechanisms underlying CO-induced arrhythmias and to define best practices for acute management and long-term follow-up.
